# Exploring Stakeholder Perceptions and Experience of Biosimilar Insulin Switching: A Scoping Review

**DOI:** 10.1002/edm2.70142

**Published:** 2025-12-30

**Authors:** Ben Hindley, Sally Wright, Cheong Ooi, Ricardo Da Costa, Louise Cope

**Affiliations:** ^1^ Clinical Pharmacy and Therapeutics Research Group, School of Pharmacy and Biomolecular Sciences Liverpool John Moores University Liverpool UK; ^2^ Pharmacy Department Liverpool University Hospitals NHS Foundation Trust Liverpool UK; ^3^ Department of Diabetes and Endocrinology Liverpool University Hospitals NHS Foundation Trust Liverpool UK

**Keywords:** Biosimilar Pharmaceuticals, drug substitution, insulin

## Abstract

**Background:**

The option to switch patients to more cost‐effective biosimilar insulins has been available since 2014, and the market share for these medicines has been slowly increasing since then. This scoping review aimed to identify the current knowledge around stakeholder perception and experience of biosimilar insulin switches.

**Methods:**

A systematic search strategy of the published literature was conducted using several bibliographic databases including PubMed, Web of Science and CINAHL Ultimate to identify relevant articles. A grey literature search and reference scouring were also employed. A thematic analysis of the literature was then conducted to identify and synthesize findings in a narrative format.

**Results:**

The search identified a total of 184 records, with 20 deemed eligible for inclusion. These comprised research studies, reviews, guidance and opinion pieces with several themes identified, including healthcare professional, patient and health service administrator perspectives. Healthcare professional concerns about switching established patients, as well as patient perceptions and experiences, were highlighted as key barriers to biosimilar insulin adoption, although patients expressing strong opinions against switching were in the minority. The established nature and proven efficacy of the reference products served as a barrier to patient acceptance. Financial considerations, especially in the context of publicly funded healthcare systems, and factors expected to facilitate biosimilar insulin switches were also identified as key themes.

**Conclusion:**

There is considerable uncertainty about how stakeholders perceive biosimilar insulin switches, particularly managed switch programmes. Almost no literature related to the experience of stakeholders who have already engaged in biosimilar insulin switching was identified. More research is needed to provide guidance on how healthcare systems can implement biosimilar insulin switch programmes in a manner acceptable to healthcare professionals and patients.

## Graphical abstract

This scoping review has systematically mapped the existing literature on stakeholder perceptions and experience of biosimilar insulin switches. It shows that there is a lack of high‐quality context‐specific research in this area, and more needs to be done to understand this topic.

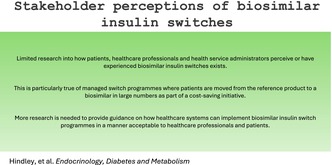

## 1 Introduction

Ensuring the most cost‐effective use of medicines is an important consideration for publicly funded healthcare systems. The relative expense of biological products and the availability of more cost‐effective biosimilars provide an attractive opportunity for significant system‐wide financial savings [1]. By increasing the uptake of biosimilar insulins, significant monetary savings can be made with theoretically no deleterious effects on the glycaemic control of insulin‐treated patients [2, 3].

A biosimilar medicine is a product that contains a highly similar version of a previously authorised biological product, usually referred to as the reference product (RP), and must undergo a stringent comparability exercise before licensing [4]. The guiding principle of biosimilar drug development is to establish similarity with the RP in terms of physiochemical properties, biological activity and clinical profile as well as excluding any difference between the products [4]. Initial in vitro and in vivo studies are followed by clinical studies to demonstrate comparability in terms of pharmacokinetic and pharmacodynamic parameters as well as comparable clinical efficacy [5]. Biosimilar medicines should have the same posology and route of administration as the RP and once licensed, it is assumed that all previously proven safety and efficacy data for the RP automatically apply to the biosimilar [3]. Table 1 outlines the biosimilar insulins that are currently available in the United Kingdom (UK) as pre‐filled pens and their price relative to the RP.

**TABLE 1:** Current UK licensed insulin products with an available biosimilar and their relative costs expressed in British pence per unit.

Insulin lispro	
Humalog KwikPen^a^	1.96p/unit^b^
Admelog SoloStar	1.47p/unit^b^ (25% price reduction)^c^
Insulin aspart	
NovoRapid FlexPen^a^	2.04p/unit^b^
Trurapi SoloStar	1.43p/unit^b^ (30% price reduction)^c^
Insulin glargine	
Lantus SoloStar^a^	2.32p/unit^b^
Abasaglar KwikPen	2.35p/unit^b^ (1% price increase)^c^
Semglee pre‐filled pen	2.00p/unit^b^ (14% price reduction)^c^

^a^ Reference product.

^b^ UK National Health Service indicative prices of disposable pen devices as per the Dictionary of Medicines and Devices on 19/07/2025.

^c^ Price difference relative to the RP.

Guidance from regulators such as the UK Medicines and Healthcare Products Regulatory Agency (MHRA), European Medicines Agency (EMA) and United States (US) Food and Drug Administration (FDA) states that biosimilars are interchangeable with their RP and that healthcare professionals (HCPs) can expect to achieve the same therapeutic effect with either product [3, 6, 7]. Furthermore, the regulators state that biosimilars are interchangeable with other biosimilars of the same RP [3, 6, 7]. Numerous studies support this position on interchangeability, having demonstrated pharmacokinetic equivalence using single‐dose euglycemic glucose clamp studies [8, 9, 10] as well as showing the comparative clinical efficacy and safety of biosimilar insulins when compared to their respective RPs [11, 12, 13, 14, 15].

Global regulators have taken differing stances on the switching and substitution of biosimilar insulins. In some territories such as the US, Canada and Australia, the practice of automatic substitution at the pharmacy level without prescriber input is permissible in certain circumstances under conditions set out by regulators [16]. This approach differs from other countries such as the UK and Japan, where switching between the RP and biosimilar must be prescriber‐directed, and the practice of substitution at the pharmacy level is not permitted [3, 16]. In the European Union, individual member states are free to decide whether the process of switching from the RP to a biosimilar requires prescriber input [16].

Given the significant financial savings that can be realised by increasing the uptake of biosimilars, coupled with their theoretical clinical equivalence, health service administrators (HSAs) have been keen to encourage their usage in preference to the RP [17]. In some countries, this has led to specific targets for biosimilar uptake in both treatment naïve and established patients [18, 19]. Assuming HCPs have confidence in the licensing of these medicines, their decision to initiate new patients on more cost‐effective biosimilar insulins does not substantially differ from the decision to use the RP, as these patients have not been previously stabilised on either product. However, advocating active switching of previously stabilised patients is entirely different, as these patients may have concerns about switching, particularly around a perceived lack of efficacy and safety [20]. Concerns such as a deficit of shared decision making, lack of choice and inadequate information have been highlighted by patient advocacy groups [21].

In addition to the concerns of patients and HCPs, there are further considerations with insulin that do not necessarily apply to other biosimilar products, such as the delivery devices, which will differ between manufacturers even if the medicine contained within is equivalent [22, 23, 24]. These differences in delivery devices can offer tangible benefits, with connected (or smart) pens able to interface with various diabetes software and allow patients to monitor the timing and dose of insulin in relation to food, exercise and blood glucose readings [22]. Such utilisation of technology is recognised as having the potential to improve patients' confidence and diabetes self‐management, which may be adversely affected if switched to a biosimilar product that does not have this functionality [22, 23]. Additionally, several insulins are also available in higher strengths for those patients with large daily insulin requirements to reduce the number and volume of injections [24]. Devices designed to deliver half‐units, primarily aimed at paediatric patients, and those that require a lower injection force are also available [25, 26]. These nuances in delivery devices are not necessarily applicable to the equivalent biosimilar, which adds an additional layer of complexity when switching patients.

Given the financial pressures faced by publicly funded healthcare systems, it is not surprising that some have undertaken mandatory biosimilar insulin switch programmes [19]. In the UK, freedom of information requests identified that at least 13 regions had undertaken some form of biosimilar insulin switch programme with a further six planning to do so. Despite such switch programmes already occurring, there appear to be deficiencies in the understanding of how HCPs, HSAs and patients view them, their willingness to participate or their experiences of them.

The aim of this scoping review was to systematically identify, map and summarise the existing literature on stakeholder perceptions and experience of biosimilar insulin switches. Table 2 outlines definitions of key terminology used as part of this scoping review for the purpose of clarity, transparency, reproducibility, as well as boundary setting. The following research question was formulated: What are the perceptions and experiences of biosimilar insulin switches from the point of view of specialist and non‐specialist HCPs, HSAs and patients?

**TABLE 2:** Definitions of terminology.

Terminology	Definition for the purpose of the review
Acceptance	The degree to which stakeholders (as defined in this table) perceive switching to a biosimilar insulin as appropriate, suitable or agreeable
Biosimilar	A product that contains a highly similar version of a previously authorised biological product and licensed as such by the regulators
Experience	Response of a stakeholder (as defined in this table) to a direct encounter with a biosimilar insulin switch event
Healthcare professional (HCP)	An individual working in the provision of health services who is currently or has previously been involved in the care of patients with diabetes in any capacity, whether as an individual practitioner or employee of a health institution or programme
Health service administrator (HSA)	An individual or body responsible for the organisation, administration or financing of healthcare services at any level
Interchangeability	The principle of switching patients from the reference product to a biosimilar (or vice versa) and achieving the same therapeutic effect
Patient	An individual of any age with insulin treated diabetes
Perception	Interpretation, understanding or belief of a stakeholder (as defined in this table) to the principle of switching patients from a reference product to a biosimilar insulin
Stakeholder	Any healthcare professional, health service administrator or patient (as defined in this table)
Substitution	The practice of switching a patient from a reference product or a biosimilar to another biosimilar of the same drug at pharmacy level without input from a prescriber

## 2 Methods

This scoping review was performed in accordance with the Arksey and O'Malley methodological framework for scoping reviews and reported in line with the Preferred Reporting Items for Systematic Reviews and Meta‐Analyses extension for Scoping Reviews (PRISMA‐ScR) [27, 28]. The protocol was developed by the research team before commencing the review and is available upon request from the corresponding author.

### 2.1 Literature Search

Three bibliographic databases (PubMed, Web of Science and CINAHL Ultimate) were searched for relevant articles related to biosimilar insulin switches in May and June 2025 and again in November 2025. These databases were chosen due to their multidisciplinary focus on biomedicine and health fields and related disciplines, which were deemed relevant to this review. The database search strategy was developed by the research team using Boolean operators along with a combination of Medical Subject Headings (MeSH) and CINAHL subject headings, along with keywords to increase the volume of literature identified. Table 3 outlines the search strategy for PubMed. A grey literature search for unpublished or more difficult‐to‐find literature was also conducted; specifically, the websites of medicines regulators and organisations responsible for healthcare strategy at a national or supranational level in the UK, Europe and North America (MHRA, EMA, FDA, US Department of Health and Human Services, National Institute for Health and Care Excellence, NHS England, NHS Scotland, NHS Wales, World Health Organisation, Health Canada) and patient advocacy groups (The Patients Association and Diabetes UK) were searched. These searches were supplemented by scanning the reference lists of the included literature to identify any further articles.

**TABLE 3:** Search strategy for PubMed.

Search	Search terms	Results
1	((“Biosimilar Pharmaceuticals”[Mesh]) AND “Insulin”[Mesh]) AND “Drug Substitution”[Mesh]	4
2	((“Biosimilar Pharmaceuticals”[Mesh]) AND “Insulin”[Mesh]) AND “Patient Acceptance of Health Care”[Mesh]	2
3	((“Biosimilar Pharmaceuticals”[Mesh]) AND “Insulin”[Mesh]) AND “Patient Satisfaction”[Mesh]	0
4	((“Biosimilar Pharmaceuticals”[Mesh]) AND “Insulin”[Mesh]) AND “Health Personnel”[Mesh]	0
5	((“Biosimilar Pharmaceuticals”[Mesh]) AND “Insulin”[Mesh]) AND “Health Services Administration”[Mesh]	18
6	((biosimilar[All Fields]) AND (insulin[All Fields])) AND (switch[All Fields])	32
7	((biosimilar[All Fields]) AND (insulin[All Fields])) AND (acceptance[All Fields])	17
8	((biosimilar[All Fields]) AND (insulin[All Fields])) AND (experience[All Fields])	14
9	((biosimilar[All Fields]) AND (insulin[All Fields])) AND (perception[All Fields])	4

### 2.2 Eligibility Criteria

Articles were included in this scoping review if they were (1) published in full text; (2) published in English; (3) published from January 2014 onwards as this date corresponds with the launch of the first biosimilar insulin; and (4) referred to the perceptions or experiences of biosimilar insulin switches of either HCPs, HSAs or patients. Quantitative, qualitative and mixed method research studies were included, as were reviews, opinion pieces and guidance or best practice documents. There were no restrictions on country of origin, patient characteristics or healthcare setting. Articles were excluded if they were (1) not available in English and in full text; (2) did not include a focus on insulin as opposed to other biological products; (3) did not examine biosimilar insulin switches; and (4) did not consider HCP, HSA or patient perspectives or experiences.

### 2.3 Study Selection and Data Extraction

Retrieved articles were stored using Microsoft Excel, and duplicate records were removed, as were any that were not written in English or where the full text was not available. Given the small number of records identified, the full text of all the remaining records was retrieved and screened by two members of the research team independently to identify those for inclusion. Any disagreements on eligibility were discussed between the reviewers before evaluation by a third member of the team if necessary. All records deemed eligible for inclusion were then imported into Mendeley Reference Manager, as well as NVivo software. The lead researcher then extracted article characteristics including the year of publication, country of origin, article type, aim and stakeholders into a data extraction tool. Any content deemed relevant to the review was highlighted using the inbuilt functionality within NVivo software.

### 2.4 Data Analysis

Once identified, the relevant content was discussed within the research team to identify and agree on key themes to which the content was then coded. This allowed for a narrative synthesis to be performed by the research team, which was presented in a narrative format.

## 3 Results

**FIGURE 1:**
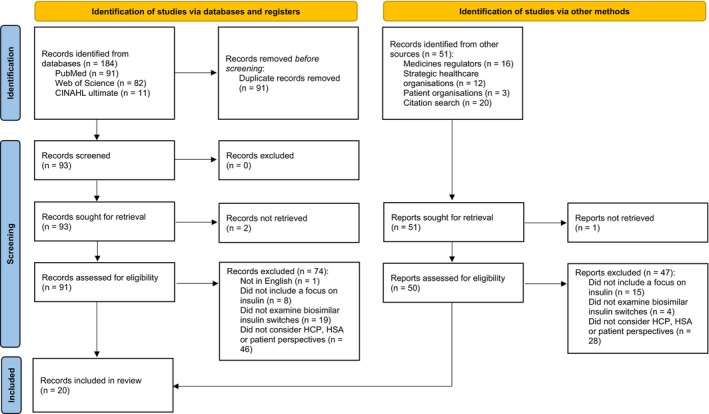
PRISMA flow diagram.

The systematic search of bibliographic databases produced 184 records, with the searches of other sources identifying an additional 51 records. After retrieval and screening, a total of 20 records were included in the review (Figure 1) [16, 29, 30, 31, 32, 33, 34, 35, 36, 37, 38, 39, 40, 41, 42, 43, 44, 45, 46, 47]. These comprised research studies (*n* = 8), guidance (*n* = 3), opinion pieces (*n* = 1) and reviews (*n* = 8), which are summarised in Table 4.

**TABLE 4:** Summary of all articles included in the review.

Author and year	Country	Article type	Article aims	Stakeholders
Joshi (2023) [16]	India	Review	Review of the current landscape of biosimilars	N/A
Dolinar (2018) [29]	US	Review	Review of biosimilar insulins	N/A
Ismail (2022) [30]	Saudi Arabia	Guidance	Inform institutional selection of biosimilars for their formulary	N/A
Jayagopal (2018) [31]	UK	Guidance	Position statement on the use of biosimilar insulins	N/A
Aladul (2019) [32]	UK	Research study	Investigate knowledge and attitudes of different HCPs towards insulin glargine biosimilars	HCPs (doctors, nurses, pharmacists)
Aladul (2018) [33]	UK	Research study	Investigate HCP's perceptions and perspectives towards biosimilar insulin glargine and the potential barriers and facilitators to prescribing	HCPs (doctors, nurses, pharmacists)
Prasanna Kumar (2024) [34]	India	Opinion piece	Opinion piece on increasing insulin access	N/A
Dowlat (2016) [35]	Germany	Review	Provide an overview of the current regulatory framework around the demonstration of interchangeability with biosimilars insulin	N/A
Barbier (2021) [36]	Belgium	Research study	Assess the knowledge and perception of biosimilars amongst HCPs	HCPs (doctors, pharmacists)
Chapman (2017) [37]	UK	Research study	Investigate HCP's knowledge and attitudes towards insulin glargine biosimilars and the factors influencing prescribing and to compare attitudes with the utilisation of these biosimilars in hospitals	HCPs (doctors, nurses, pharmacists)
Vandenplas (2021) [38]	Belgium	Review	Review of the current market landscape of off‐patent biosimilars	N/A
Wilkins (2014) [39]	US	Research study	Identify acceptance of a hypothetical future switch to a less expensive biosimilar insulin	Patients
Shubow (2022) [40]	US	Review	Review of the experiences of academic clinicians and their perspectives on how to increase biosimilar adoption	N/A
Fisher (2022) [41]	Canada	Research study	Identify early changes in health services utilisation after a mandatory policy to switch patients from originator to biosimilar insulin glargine	Patients
Taki (2020) [42]	Japan	Research study	Evaluate insulin treatment satisfaction, safety and effectiveness of biosimilar insulin glargine in patients with type 2 diabetes who switched from originator insulin glargine	Patients
Abitbol (2025) [43]	Canada	Review	Review of the efficacy, safety, regulatory and health economic considerations of biosimilar insulins	N/A
Polimeni (2015) [44]	Italy	Review	Review of the issues concerning biosimilar insulins	N/A
White (2022) [45]	US	Review	Review of the current US diabetes policy landscape and the differences between biosimilar insulins and follow‐on insulins and considerations for successful adoption of biosimilar insulins	N/A
Diabetes UK (2019) [46]	UK	Guidance	Position statement on the use of biosimilar insulins	N/A
Car (2025) [47]	Europe	Research study	Explore the key factors influencing HCP's prescribing decisions regarding biosimilar and RP insulin	HCPs (doctors)

### 3.1 HCP Perspectives and Experience of Switching

Four of the research studies found that awareness of the principles of biosimilars amongst HCPs was high, particularly in relation to their understanding that biosimilars are copies of another biological medicine (the RP) [32, 33, 36, 37]. These studies also found that pharmacists demonstrated the highest level of awareness, although a lack of training on biosimilars limited their confidence in leading discussions with patients [32, 36]. Despite the high level of awareness across professions, 67% of surveyed HCPs lacked practical experience of switching patients, potentially impacting negatively on patient acceptance [36, 43]. A study critiquing prescribing practice found that diabetologists were less frequent prescribers of biosimilars, with biosimilar insulin glargine representing only 9% of utilisation, compared to gastroenterologists and rheumatologists' utilisation of biosimilar infliximab, which was 67% and 39%, respectively [37].

The level of concern expressed by HCPs varied across research studies, with one finding that amongst diabetologists, 25% expressed major concerns in relation to safety and 32% in relation to efficacy when initiating new patients on biosimilars [37]. Another study found similar levels of major concern amongst doctors (safety 14%, efficacy 22%) and pharmacists (safety 19%, efficacy 16%), but concern amongst nurses was higher (safety 42%, efficacy 54%) [32]. When asked about switching patients, levels of concern amongst the HCPs increased, reaching as high as 63% for nurses when asked about efficacy, but were lower for doctors (safety 28%, efficacy 34%) and pharmacists (safety 38%, efficacy 50%) [32, 37]. Concerns amongst diabetologists about both initiating and switching were significantly higher than that expressed by other specialities, specifically gastroenterology, where fewer than 10% expressed major concerns [37].

Although only a minority of HCPs surveyed (32%) believed that there was insufficient data to support switching, both research studies and opinion pieces found that questions about the interchangeability of biosimilar insulins were highlighted as having the potential to impede widespread use [34, 36]. The lack of long‐term safety data and potential for the development of unexpected side effects were cited in both research studies and reviews as barriers to prescribing, as was the perception that biosimilars are less clinically tested [33, 35, 36, 40]. The narrow therapeutic index of insulin was also raised in reviews as a potential issue, especially given the hypoglycaemic risk [35, 44]. Another review suggested that a lack of knowledge around the specifics of biosimilar regulatory approval may have impeded prescribing [40].

Some organisational guidance recommended against switching established patients to a biosimilar, especially when glycaemic control was satisfactory, as mandated switches may not fully consider the needs of individual patients with diabetes [31, 43, 46]. Qualitative studies have offered support for this, with respondents stating that patient opinion would prevent them from switching and that patients should have the right to refuse an automatic switch [33, 47]. Despite this, there was concern among some HCPs that they were under organisational pressure to use biosimilars [33].

One research study highlighted questions from patients about efficacy, safety, immunogenicity, similarity and interchangeability, as well as the method of administration, as key challenges to dispensing biosimilars [36]. Almost all (95%) of pharmacists expressed a desire for further training [36]. Some HCPs also highlighted the challenge of stocking multiple biosimilar versions of the same medicine [40].

A number of research studies and reviews highlighted that HCPs considered the switching of biosimilars without the intervention of the prescriber, a process known as substitution, to be a highly contested point and that a prescriber should be fully aware of any changes in case of issues with safety or response [29, 33, 36, 45]. It was also highlighted that this approach could result in multiple switches between biosimilars, an area where there was a paucity of data, and potentially reduce levels of patient trust [16, 33]. The challenge of linking adverse events to one particular product following repeated switches was also emphasised, as was the need for additional data to determine interchangeability [35, 36, 44]. In addition to pharmacy‐led substitution, some HCPs also expressed disagreement with the idea of generalist prescribers switching patients, believing that any switch requires review and ongoing supervision by specialists [31, 36]. One review concluded that the practice of substitution was perceived by some HCPs as an infringement upon their decision‐making power [40].

The switching of devices was highlighted in both reviews and guidance as having implications for HCPs in terms of the workload associated with training and monitoring, especially given that devices differ [29, 31]. Additionally, there were concerns that patient acceptance and compliance were highly dependent on the delivery device [35, 47]. Both UK and Belgian studies found that HCPs perceived that biosimilar switching would have a negative impact on departmental resources [33, 36].

Overall, although awareness was high, HCPs expressed some concerns related to biosimilar insulins, particularly around switching existing patients and the potential for negative outcomes related to the change in medicine or device. There was also skepticism around pharmacy‐level substitution. Several barriers, including training, workload, differences in delivery devices, and a lack of long‐term data, also appeared to be limiting uptake among diabetologists. No literature was identified regarding the experiences of HCPs who had participated in biosimilar insulin switches or how experiences or perceptions differed between professions.

### 3.2 Patient Perspectives and Experience of Switching

Only three research studies considering patient perceptions from the US, Canada and Japan were identified [39, 41, 42]. The single research study from the US examining patient perspectives about a theoretical future switch to a biosimilar insulin found that only 5% of those with type 1 diabetes indicated that they would definitely not use a biosimilar, with a further 15% stating that they would be unlikely to use one [39]. This was slightly higher than in those patients with type 2 diabetes (4% and 11%, respectively) [39]. The majority of those asked were either indifferent or positive about the potential of switching to a biosimilar insulin, overall, with 66% of those asked expressing positive viewpoints about the idea [39].

Reasons for not wanting to switch to a biosimilar insulin included a perception of brand quality, satisfaction with current therapy, substantial differences in research and investment between the RP and biosimilar, and the proven track record of the RP [39]. Previous negative experiences with generic switching were also a barrier to patient acceptance [39].

Patients raised concerns related to comparative effectiveness between biosimilar insulins and their RP, specifically whether it would be as effective as their current therapy [35, 39]. Other concerns highlighted included issues such as absorption rate, storage requirements, stock availability and the oversight of the manufacturing process [39]. Reviews also highlighted that the potential for side effects and changes in delivery devices were also discouraging for patients, with many asking specifically about the availability of pen devices [29, 35, 40].

In countries where patients were required to cover some or all of the cost of their medicines, such as the US, price was seen as the biggest factor in increasing uptake, especially for those who pay out of pocket, but was less of an issue for those where their insurance covered all or most of the costs [39, 45]. Studies found that financial incentives had a large impact on acceptance, although some patients associated the lower cost of biosimilars with reduced quality [39, 41]. One review suggested that the availability and affordability of biosimilar insulins could increase adherence to treatment [16].

A single Japanese study of patients who had experienced a switch from reference to biosimilar insulin glargine found that there was overall no change in treatment satisfaction, efficacy or adverse events [42]. A further research study from Canada examining the impact of a mandatory non‐medical switch to biosimilar insulin glargine found that only 2.8% of patients subsequently switched back to the RP [41].

Overall, although there was limited data related to patient perceptions or experiences, what was available did suggest that patients were broadly supportive of the principle of biosimilar insulin switches.

### 3.3 HSA Perspectives and Experience of Switching

Guidance for HSAs was more focused on the administrative aspect of biosimilar insulin switching. Selecting a particular biosimilar, especially when several were available, and the question of whether to also utilise the RP was a concern of HSAs [30]. The need to be certain about the regulatory status of the biosimilar was also raised [30].

Both reviews and guidance concluded that HSAs require a clear organisational plan for switching as well as guidelines, inclusion and exclusion criteria, a plan for education of HCPs and patients especially in the event of device changes, and how to handle certain vulnerable subpopulations [16, 30, 43]. The importance of safe transition of care and concerns over negative impressions from prescribers leading to a nocebo effect were also raised [30].

Additionally, guidance highlighted concerns over the legal responsibilities of switching patients, resulting in some organisations favouring a shared decision‐making and informed consent process for switching [30].

One study assessing changes in healthcare resource usage following mandatory switching of patients to biosimilar insulin glargine found no evidence of an increase in patient demand for healthcare resources [41].

### 3.4 Financial Considerations When Switching to Biosimilar Insulins

In countries where governments played a more active role in price regulation, such as the UK, HCPs were sensitive to the need for financial savings and felt that cost was the dominant consideration when prescribing biosimilars, but that a minor price difference between products would act as a disincentive [30, 33, 37, 38]. One UK study found that hospital‐based HCPs were reluctant to switch patients due to the relatively short time spent with these patients and a perceived lack of budgetary benefit, stating that any switching should be done in the community as they are the financial beneficeries [33]. In a community care setting, HCPs again expressed the need for financial incentives to stimulate prescribing, particularly increased staffing to manage biosimilar implementation [38].

In countries where patients covered some or all of the cost of their medicines, such as the USA and Canada, because of the high cost of biologics and pressure from payors, reviews concluded that HCPs may be forced to switch to using less expensive options [29, 30, 43]. Despite the lower cost of biosimilars, some HCPs were concerned that savings would not be fully passed onto the patients with insurance providers being the primary beneficiary [40].

In addition to the financial incentives, a UK study concluded that HCPs felt that biosimilars were important to stimulate competition between manufacturers, which would lead to downward pressure on prices [37].

### 3.5 Factors Expected to Facilitate Switching

The most frequently cited factors in research studies and reviews across several countries related to evidence base and education. HCPs cited robust scientific data and post‐marketing pharmacovigilance studies as the most important influence on increasing uptake, as well as support from national policy and professional bodies, other healthcare providers, local policies and structured initiatives to increase usage [32, 33, 36, 37, 40, 44]. Compelling evidence from interchangeability studies and confidence in the regulators' evaluation processes were also desirable, as was ensuring that the biosimilar had been clinically tested for the same indications [34, 35, 36]. Compelling real‐world evidence was also deemed to have a significant positive effect on uptake [34, 35, 40, 45]. Clear guidance on practicalities such as dosing was also mentioned [30]. Educational initiatives aimed at HCPs to improve prescribing confidence alongside those aimed at patients were cited in both reviews and guidance as important to increase usage of biosimilars [30, 34, 40, 45]. The authors of one study concluded that incorporating teaching on biosimilars into undergraduate degree courses could serve to increase uptake and support for switching programmes [36].

Some reviews and guidance stated that seeking informed consent, providing positive framing and reassurance, as well as including patient advocates on decision‐making committees, were all positive factors [16, 30]. Authors of both a research study and review concluded that providing the option for patients to switch back to the RP might increase uptake by improving HCP engagement [33, 40].

Similarity in delivery devices between manufacturers was perceived to be a positive, and some HCPs believed that poor glycaemic control provided a good opportunity to discuss switching as part of management [29, 30, 31, 33].

Regarding financial savings, clear information on whether this would be reinvested locally was mentioned as a key facilitator in two UK research studies [32, 37]. Given that switching patients requires effort, reimbursement for this work was highlighted as an important factor in one Canadian review [43]. In areas where patients covered the cost of some or all of their treatment, the authors of one review concluded that prescriber decisions may be based on the patient's ability to pay, making switching more common [16].

It was suggested in both a research study and an opinion piece that allowing substitution at the pharmacy level could simplify and streamline the process of switching patients [34, 36].

## 4 Discussion

This scoping review achieved the stated aim of systematically identifying, mapping and summarising the existing literature on stakeholder perceptions and experience of biosimilar insulin switches and is the first to do so. The need for greater understanding has become more important given the increased likelihood of managed switch programmes becoming commonplace as a means for healthcare systems to make financial savings [48].

The very limited available literature on the perspectives of HCPs identified that most involved in the care of patients with diabetes were willing to both initiate biosimilars in insulin naïve patients as well as switch existing patients, although concerns were understandably higher with respect to switching [32, 33, 37]. There was no data regarding how opinions differed between different professions.

One finding was that some of the perceived barriers to the prescribing of biosimilars included the lack of long‐term safety data and clinical testing when compared to the RP [33, 35, 36, 40]. Although a legitimate concern, this does not align with the guidance from regulators that all previously proven safety and efficacy data for the RP automatically apply to the biosimilar, meaning that, by default, biosimilars have the same level of safety and efficacy data as the RP [3, 6, 7]. This would suggest either a lack of understanding of the principles of biosimilar drug development or skepticism of the licensing process.

Although the literature identified that few HCPs believe that there is insufficient data to support switching, the actual or perceived lack of real‐world data is potentially hindering the uptake of biosimilar insulins, and the existence of such evidence was highlighted as a factor influencing prescribing [34, 35, 36, 40, 45]. The licensing of biosimilar insulins requires robust evidence of similarity in terms of pharmacokinetic profile, efficacy and safety [3]. Despite this, concerns that glycated haemoglobin alone may be a poor indicator of clinical efficacy and fail to capture the impact of blood glucose variations on patients' quality of life have the potential to reduce trust, especially given that the licensing of biosimilar insulins is heavily dependent on this one marker of efficacy [49, 50].

When compared to other specialties such as gastroenterology, diabetologists appear to have a lower level of acceptance of biosimilar switches [37]. The reasons for this are unclear and may be due to their level of experience, the setting in which these medicines are used (i.e., infusions of biologics in a managed hospital setting vs. self‐administration in the community), or the magnitude of potential financial savings [51]. Diabetologists may also be more reluctant to switch patients out of concerns related to adverse effects such as severe hypoglycaemia or loss of efficacy, which can be difficult to manage in the ambulatory care setting. Differences in delivery device functionality may lead to HCPs favouring certain manufacturers' products and reluctance to recommend a biosimilar that lacks such functionality. This is an important consideration given the tangible benefits some devices offer, such as the ability to interface with diabetes software, deliver half units or reduce the number of injections required [22, 23, 24]. Additional considerations around paediatric patients' willingness to move to an unfamiliar product or the ability of vulnerable patient groups to safely transition to a new device may also be impairing biosimilar insulin switches and have not been adequately researched. The clinical implications of lower acceptance of biosimilar switching among diabetologists are unclear but have the potential to reduce access to more affordable alternative insulins in some jurisdictions, as well as limiting competition between manufacturers.

All the studies examining HCP perceptions were entirely theoretical, with no literature detailing involvement or experience of switching patients either individually or as part of a managed switch programme.

This scoping review identified a paucity of information regarding patient perspectives of switching to biosimilar insulins. The single study identified that focused specifically on patient perspectives was conducted in the USA in 2014, and although it showed generally high levels of acceptance about the prospect of switching to a biosimilar insulin, it is questionable how applicable this is to patients in countries with publicly funded healthcare systems [39]. A financial incentive exists for patients to switch to a more cost‐effective product when they are partly or fully responsible for covering the cost, and unsurprisingly, those patients whose costs were mostly or fully covered by insurance were less keen on switching [39].

Unlike HCP perceptions, there was some real‐world evidence in relation to patients' lived experience of switching, but the amount of literature was limited [41, 42]. One study that did investigate patient satisfaction before and after a biosimilar insulin switch found that patients were generally happy with the process [42]. The data regarding patients switching back to the RP from a biosimilar was also sparse, with only one study identified [41]. Although the study did find that the numbers of patients who reverted to the RP following a mandated switch to a biosimilar were low, the patient cohort had a strong financial incentive to remain on the biosimilar as their insurance provider had ceased to cover the RP, again raising questions over how applicable this finding is for countries with publicly funded healthcare systems. None of the studies explored patient perspectives in publicly funded healthcare systems where the cost of the medicine is not a direct patient concern.

HSAs concerns centred around the administrative aspect of the process, specifically legal responsibilities, the selection of biosimilars for the formulary and the need for clear guidance and processes to manage switches, as this is something that would fall within their remit [30]. The general lack of evidence in support of managed switch programmes has the potential to reduce their efficacy, as they are unlikely to be able to demonstrate a strong evidence base in support of their methodology, and HCPs may be reluctant to switch patients, thwarting its success [30]. Additionally, given the high‐risk nature of insulin and the nuances of insulin delivery already discussed, without robust evidence to inform the development of such programmes, there is a greater risk of unintended adverse outcomes for patients.

The literature highlighted the need for at least part of any financial gains to be realised by the teams responsible for switching patients as recognition for the work undertaken [33, 38]. The use of financial incentives and gain sharing has been shown to increase biosimilar uptake in other specialities [52].

Strong support from guideline developers and professional bodies was also highlighted as a key driver for increasing uptake, which was expected given the influence of such organisations over practice [32, 33, 36, 37, 40]. Although some national guideline developers did endorse biosimilar insulin switches, they highlighted the need for discussions with individual patients in line with the principles of shared decision making, which falls considerably short of endorsement of any managed switch programme [53, 54]. The position of some patient advocacy groups is that patients should not be switched ‘without good clinical reason’ which could create some conflict with those responsible for funding healthcare [46, 55, 56]. It may therefore be time to consider updating guidance to more expressly support the managed switching of patients to more cost‐effective biosimilars.

Another consideration was that in the future, as more biosimilar insulins come to market, it may not be practical for organisations to manage repeated switches from both a workload and patient acceptance point of view. One alternative would be to adopt the approach of those regulators that allow substitution at the pharmacy level without the need for any direct input from a prescriber, but questions about HCP, patient and HSA perspectives must be answered before any such departure from established clinical practice [16].

In conclusion, there is considerable uncertainty about how HCPs, HSAs and patients perceive or have experienced biosimilar insulin switches, particularly managed switch programmes. There is also uncertainty on how opinions, experiences or perceptions differ between HCPs who specialise in diabetes and those who do not. Issues such as perceived or actual workload, clinician confidence and patient acceptance, particularly when being asked to swap from the RP on which they have previously been stabilised onto a biosimilar, do not appear to have been adequately researched. The lack of qualitative studies exploring patient experiences represents a significant limitation for informing policy decisions. A summary of the key topics discussed and how they might be addressed is available in Table 5.

**TABLE 5:** Summary of the topics highlighted and how they might be addressed.

Topics highlighted	Potential methods to address the issues raised
Limited research on patient experience of biosimilar insulin switches, especially managed switch programmes and how experience differs between different groups, geographical locations or healthcare systems	Undertake high‐quality context specific research in participants who have experienced a biosimilar insulin switch
Perceived lack of long‐term data and perceived lack of clinical testing amongst HCPs	Improved education around the principles of biosimilar drug development to provide reassurance, coupled with outcome data from research examining patient and HCP experience of biosimilar insulin switches
Lower level of acceptance amongst diabetologists	Undertake further research in this group of healthcare practitioners to identify the underlying reasons for this
Limited research to support HSAs to deliver successful evidence‐based switch programmes	Undertake high‐quality context‐specific research in participants who have participated in managed switch programmes and develop best practice guidance

The primary limitation of this scoping review was the very small amount of research that has been conducted in relation to biosimilar insulin switches, especially from the perspective of patients. The literature identified was mostly limited to higher‐income countries, limiting the applicability of the review's findings to low and middle‐income jurisdictions. Some of the literature dates from 2014 to 2018 when biosimilar insulin adoption was in its infancy, and it is possible that stakeholder perceptions may have evolved since then. Additionally, some of the literature identified did not focus specifically on biosimilar insulin switches and often included other biosimilar products. As a scoping review with a diverse mix of article types, a critical appraisal of the evidence was not undertaken, which limited the ability of the authors to assess the reliability of the evidence. However, given the small amount of available literature and the robust literature search, it is likely that all or most of the relevant published articles were identified.

High quality, context‐specific research is urgently needed to answer questions about stakeholder experience of biosimilar insulin switches and support healthcare systems to effectively balance the needs of individual patients with diabetes and the need to make financial efficiencies.

## Author Contributions

**Ben Hindley:** conceptualization, investigation, writing – original draft, methodology, writing – review and editing, formal analysis, data curation. **Sally Wright:** writing – review and editing, formal analysis, supervision, methodology. **Cheong Ooi:** conceptualization, writing – review and editing. **Ricardo Da Costa:** investigation, writing – review and editing. **Louise Cope:** writing – review and editing.

## Funding

The authors have nothing to report.

## Conflicts of Interest

Two of the authors have been involved in a biosimilar insulin switch programme in the UK. The other authors declare no conflicts of interest.

## Data Availability

The authors have nothing to report.
